# Causes of variability in latent phenotypes of childhood wheeze

**DOI:** 10.1016/j.jaci.2018.10.059

**Published:** 2019-05

**Authors:** Ceyda Oksel, Raquel Granell, Osama Mahmoud, Adnan Custovic, A. John Henderson, the STELAR, Breathing Together investigators

**Affiliations:** a Section of Paediatrics, Department of Medicine, Imperial College London, London, United Kingdom; b Department of Population Health Sciences, Bristol Medical School, University of Bristol, Bristol, United Kingdom

**Keywords:** Childhood asthma, wheeze phenotypes, longitudinal analysis, latent class analysis, Avon Longitudinal Study of Parents and Children, ALSPAC, Avon Longitudinal Study of Parents and Children, ARI, Adjusted Rand index, BIC, Bayesian information criterion, LCA, Latent class analysis, TCRS, Tucson Children's Respiratory Study

## Abstract

**Background:**

Latent class analysis (LCA) has been used extensively to identify (latent) phenotypes of childhood wheezing. However, the number and trajectory of discovered phenotypes differed substantially between studies.

**Objective:**

We sought to investigate sources of variability affecting the classification of phenotypes, identify key time points for data collection to understand wheeze heterogeneity, and ascertain the association of childhood wheeze phenotypes with asthma and lung function in adulthood.

**Methods:**

We used LCA to derive wheeze phenotypes among 3167 participants in the ALSPAC cohort who had complete information on current wheeze recorded at 14 time points from birth to age 16½ years. We examined the effects of sample size and data collection age and intervals on the results and identified time points. We examined the associations of derived phenotypes with asthma and lung function at age 23 to 24 years.

**Results:**

A relatively large sample size (>2000) underestimated the number of phenotypes under some conditions (eg, number of time points <11). Increasing the number of data points resulted in an increase in the optimal number of phenotypes, but an identical number of randomly selected follow-up points led to different solutions. A variable selection algorithm identified 8 informative time points (months 18, 42, 57, 81, 91, 140, 157, and 166). The proportion of asthmatic patients at age 23 to 24 years differed between phenotypes, whereas lung function was lower among persistent wheezers.

**Conclusions:**

Sample size, frequency, and timing of data collection have a major influence on the number and type of wheeze phenotypes identified by using LCA in longitudinal data.

Wheeze is a common symptom in the early years of life, with nearly one third of children experiencing it at least once before their third birthday.^1^, ^2^, ^3^ Although the symptoms of most infants with wheeze seem to remit by the time the child reaches school age,^4^ infantile wheeze can also persist into later childhood and adulthood after a period of remission.^5^, ^6^ Conversely, the majority of patients with persistent asthma start wheezing in early childhood.^2^ However, at the onset of symptoms, patients with “transient wheeze” and “persistent wheeze” look very similar, and it is difficult to predict which of the early childhood wheezers will stop wheezing (and when) and which will have persistent wheezing and asthma.

Understanding the heterogeneity of wheezing disorders and distinguishing wheeze phenotypes in early childhood is critical to developing interventions targeted at those who will persist with wheezing into later childhood and to avoid overtreatment of patients with transient wheeze.^7^ Over the last 2 decades, substantial effort has been devoted to understanding the heterogeneity of childhood wheezing illness.^7^, ^8^, ^9^, ^10^ In general, population-based birth cohorts are regarded as optimal data sources for understanding temporal patterns of wheezing and relating them to different risk factors because the information is collected prospectively and therefore free from recall bias.^11^

The initial approach of hypothesis testing using data on wheezing collected at the ages of 3 and 6 years in the Tucson Children's Respiratory Study (TCRS) described 3 wheeze phenotypes: transient early, late onset, and persistent.^2^ This finding was confirmed in several independent cohorts.^3^, ^12^, ^13^ Subsequently, the methodology to discover “wheeze phenotypes” was extended to the use of unsupervised data-driven approaches, such as latent class analysis (LCA).^1^, ^14^, ^15^, ^16^, ^17^, ^18^ These analyses revealed a different structure within the data and suggested the existence of 1^19^, ^20^ or 2 further intermediate phenotypes.^1^, ^17^, ^18^ It is important to emphasize that although wheeze phenotypes derived from different analyses tend to share the same nomenclature, phenotypes with the same assignment often differ substantially in terms of the age of onset, temporal trajectory, distributions within a population,^8^ and associated risk factors, making comparison between studies difficult and clinical application uncertain.^8^, ^10^ For example, late-onset wheezers were reported to start experiencing symptoms after the age of 3,^19^ 4,^16^ or 5^13^ years in different studies. Inconsistencies between studies can be partly attributed to differences in study design or could be due to true differences between different populations. However, this seems unlikely because most evidence comes from broadly similar population-based studies with comparable ethnic mixes.

If we are to understand factors associated with patterns of wheezing with different long-term consequences, then phenotypes must be consistent and reproducible. Despite the widespread use of LCA, little is known about the external factors that influence the outcomes of LCA models in phenotype identification. We propose that sample size and the timing and frequency of data collection affect the number and type of discovered wheeze phenotypes in LCA and that not all time points carry useful information (and therefore some might be redundant or even cause uncertainty in the results).

To provide a better understanding of the influence of input data characteristics on the identified longitudinal trajectories of wheezing, we investigated the effect of the number of data points, age at which information was collected, and sample size on the number and/or nature of wheeze phenotypes discovered by LCA. We also sought to identify data collection points, which are most informative in distinguishing wheeze phenotypes.

## Methods

### Study design, setting, and participants

The Avon Longitudinal Study of Parents and Children (ALSPAC) is a population-based birth cohort established in 1991 in Avon, United Kingdom. It recruited 14,701 children born between April 1, 1991, and December 31, 1992. Ethical approval for the study was obtained from the ALSPAC Ethics and Law Committee and local research ethics committees. Details of the study protocol can be found elsewhere.^21^ The study Web site contains details of all the data that are available through a fully searchable data dictionary at www.bris.ac.uk/alspac/researchers/data-access/data-dictionary/.

### Data sources and definition of outcomes

Participating mothers were sent a self-completion questionnaire about the health of their children at 14 time points from birth to age 16½ years: months 6, 18, 30, 42, 57, 69, 81, 91, 103, 128, 140, 157, 166, and 198. Current wheezing was defined as a positive answer to the following question: “In the last 12 months has he/she had any periods when there was wheezing or wheezing with whistling on his/her chest when he/she breathed?”^22^

Study subjects attended a research clinic at age 23 to 24 years in which lung function was measured by using spirometry.^23^, ^24^ Postbronchodilator FEV_1_ was ascertained 15 minutes after administration of 400 μg of salbutamol. We expressed FEV_1_ as percent predicted values against Global Lung Function Initiative curves.^25^ *Self-reported asthma ever* was defined as a positive answer to the following question: “Have you ever had asthma?” *Self-reported current asthma* was defined at age 23 years as asthma ever together with a positive answer to either of the following questions: “Have you had any wheezing or whistling in the past 12 months?” or “Have you taken asthma medication in the last 12 months?”

### Statistical analysis

Children with complete reports of wheezing at all 14 time points from birth to age 16½ years (n = 3167) were included in the analysis to obtain better representation of the latent structure. We performed LCA to investigate how latent class subpopulation structure varied by the timing and frequency of observations. Starting with a latent model including 4 phenotypes, we compared models with varying sample sizes (3167, 2500, 2000, 1500, 1000, and 500), numbers of latent classes (4-6), and numbers of time points (14, 11, 8, and 6) based on their statistical fit, including the Akaike information criterion, Bayesian information criterion (BIC), Lo–Mendell–Rubin and bootstrapped likelihood ratio, model quality (model entropy), and interpretability. The best-fitting model in each run was selected based on the lowest BIC. We then repeated our analyses among 12,290 participants with at least 2 questionnaire responses. We identified critical data collection points for identification of distinct phenotypes of wheezing based on stochastic evolutionary search through a genetic algorithm (see the Methods section in this article's Online Repository at www.jacionline.org for more details on the methodology for selection of informative data collection points). The adjusted Rand index (ARI) was used as a similarity measure when comparing different clustering results. Variable specific entropy values were used to show how well individual data collection points identify the latent classes. We calculated CIs for the difference of population proportions to compare the frequency of participants with asthma at age 23 years between different phenotypes. Differences in lung function were tested by using 1-way ANOVA and the Tukey honestly significant difference test. All analyses were performed in Stata software (version 15), Mplus 8, and R software by using the packages poLCA,^26^ DiagrammeR, and LCAvarsel.^27^

## Results

A total of 3167 participants had complete reports of wheeze at all 14 time points. In line with our previous results,^17^, ^18^ the best-fitting model resulted in 6 distinct wheeze phenotypes: never/infrequent wheezing; persistent wheezing; 2 early-onset transient classes (early-onset preschool remitting and early-onset midchildhood remitting); and 2 late-onset persisting classes (school-age onset and late-childhood onset).

### Influence of sample size

**Fig 1:**
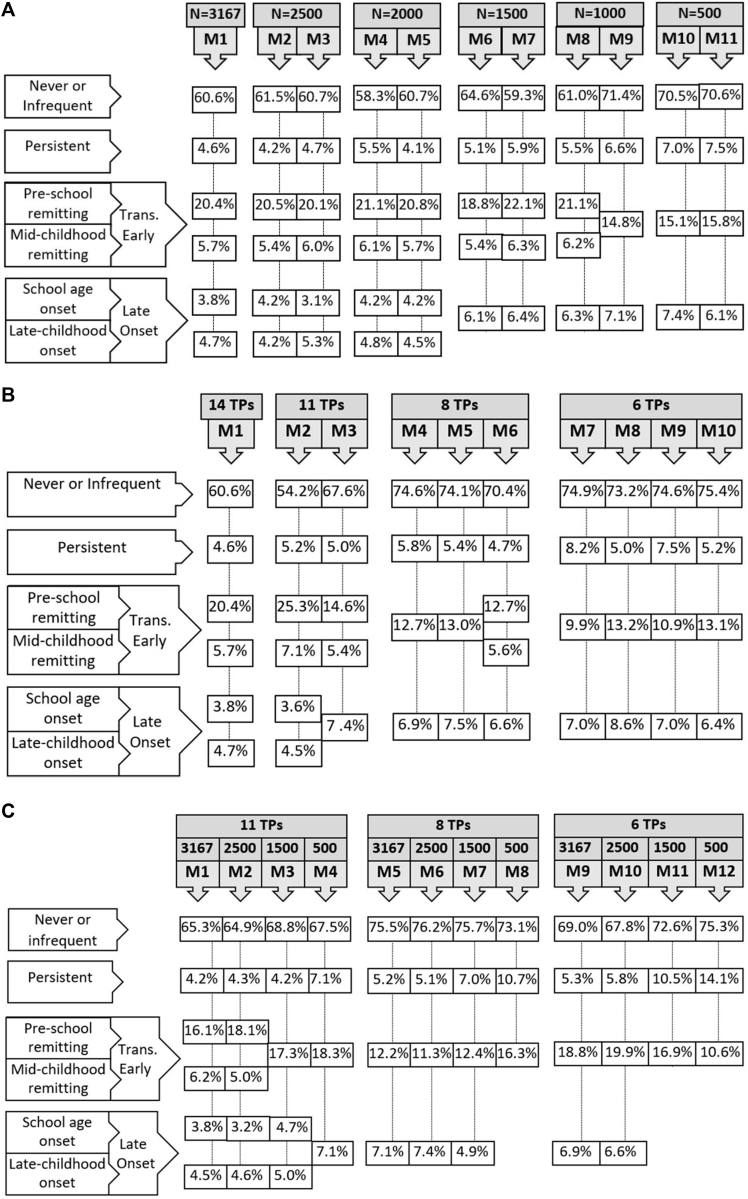
Optimal number, shape, and prevalence of wheeze phenotypes identified by using LCA. **A,** Eleven latent models based on randomly selected subsamples of 6 different sample sizes (n = 500, 1000, 1500, 2000, 2500, and 3167) while maintaining a constant number of follow-up points (14 time points). **B,** Ten latent models based on randomly selected time points (6, 8, 11, and 14 time points) while maintaining a constant sample size (n = 3167). **C,** Twelve latent models based on randomly selected subsamples of 4 different sample sizes (n = 500, 1500, 2500, and 3167) and different numbers of time points (6, 8, and 11 time points).

We varied the sample size from 3167 to 500 and developed 11 different models based on randomly selected subsamples of 6 different sizes (n = 500, 1000, 1500, 2000, 2500, and 3167), holding all else constant. Fig 1, *A*, shows the best-fitting models based on different sample sizes and the prevalence of each phenotype based on the estimated model. Four phenotypes (never/infrequent, persistent, transient early, and late onset) were identified with a sample size of 500. The best-fitting model based on 1000 participants resulted in 4 to 5 phenotypes.

**Fig E1:**
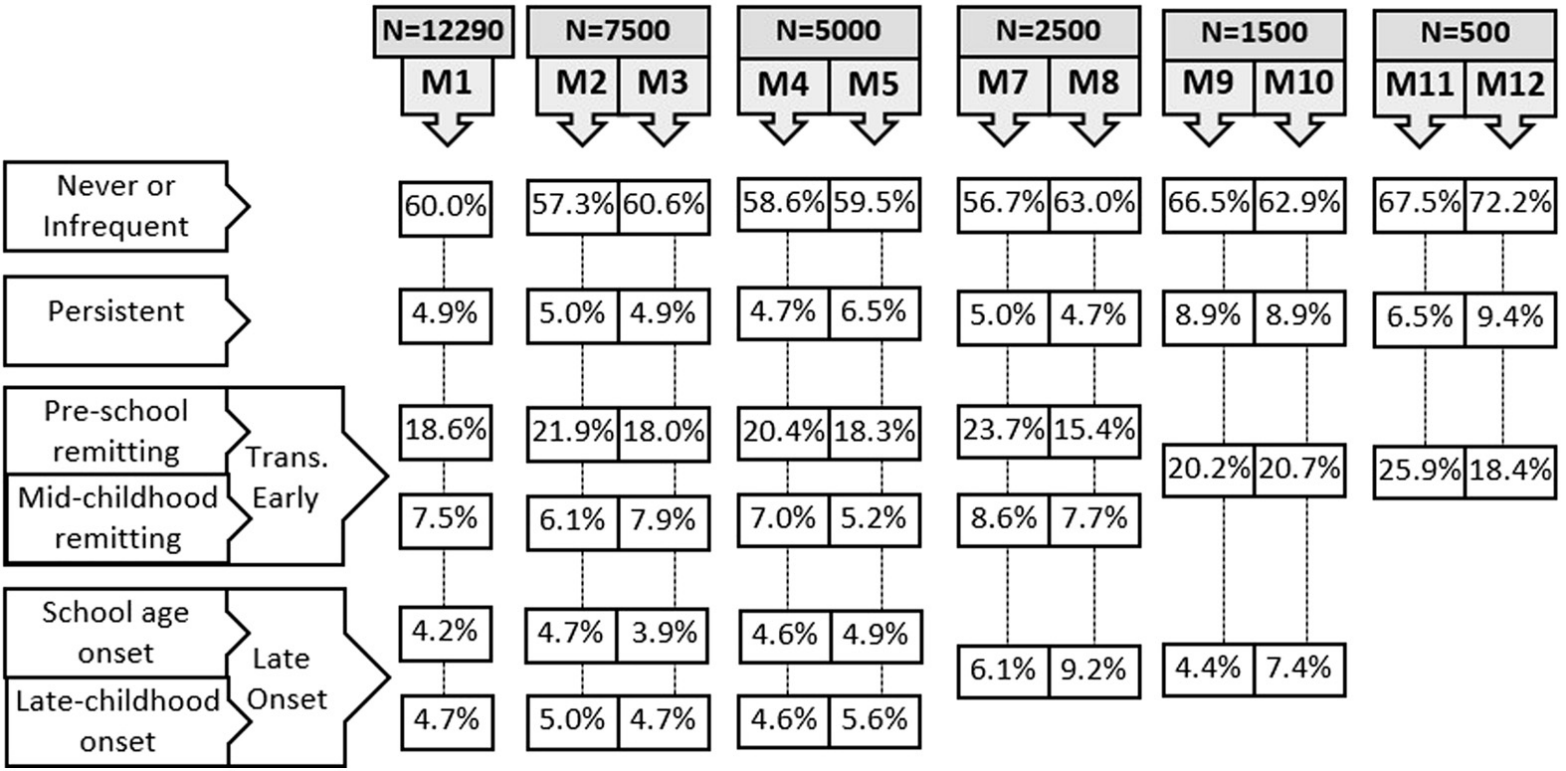
Optimal number, shape, and prevalence of wheeze phenotypes with different (12,290, 7,500, 5,000, 2,500, 1,500, and 500) sample sizes based on children with at least 2 observations of wheezing (the optimal phenotype was chosen based on the lowest BIC).

Larger sample sizes (≥2000 participants) were needed to detect smaller phenotypes (<5% frequency). LCA identified 6 latent wheeze phenotypes in samples of 2000 or more children with complete data (Fig 1, *A*) and in samples of 5000 or more children with incomplete data (see Fig E1 in this article's Online Repository at www.jacionline.org).

### Influence of data collection frequency

We then varied the frequency of data collection time points from 6 to 14 and developed 10 different models based on randomly selected time points while maintaining a constant sample size (n = 3167). Adding more time points to the latent model increased the number of wheeze phenotypes that were identified (Fig 1, *B*). However, in some cases an identical number of (randomly selected) data collection points (eg, 11 time points) resulted in different optimal numbers of phenotypes, depending on intervals between time points. This suggests that, in addition to sampling frequency, timing and distribution of time points at which data are collected can influence wheeze phenotype identification and that there might be critical data collection points that are more informative in distinguishing wheeze phenotypes.

### Combined effects of sample size and data collection frequency

To examine how both the frequency of data collection (number of time points) and the size of the studied population affects the optimal number, trajectory, and frequency of the identified phenotypes, we varied the number of data collection points from 6 to 11 and randomly selected subsamples of 4 different sizes, resulting in a total of 12 data conditions (Fig 1, *C*).

Models with small sample sizes (n < 2500) did not identify low-frequency phenotypes (<5%), regardless of the frequency of data sampling. However, there was a clear link between sample size, number of data points, and optimal number of wheeze phenotypes. Models with sample sizes of 2500 or greater identified 6 phenotypes when the number of data collection points included in the analysis was relatively high. However, models with decreasing numbers of data points were unable to detect 6 phenotypes, and models with the same sample size did not identify small phenotypes (<5% frequency) under certain conditions (eg, number of time points < 11).

### Selection of the most informative data collection points

**Fig E2:**
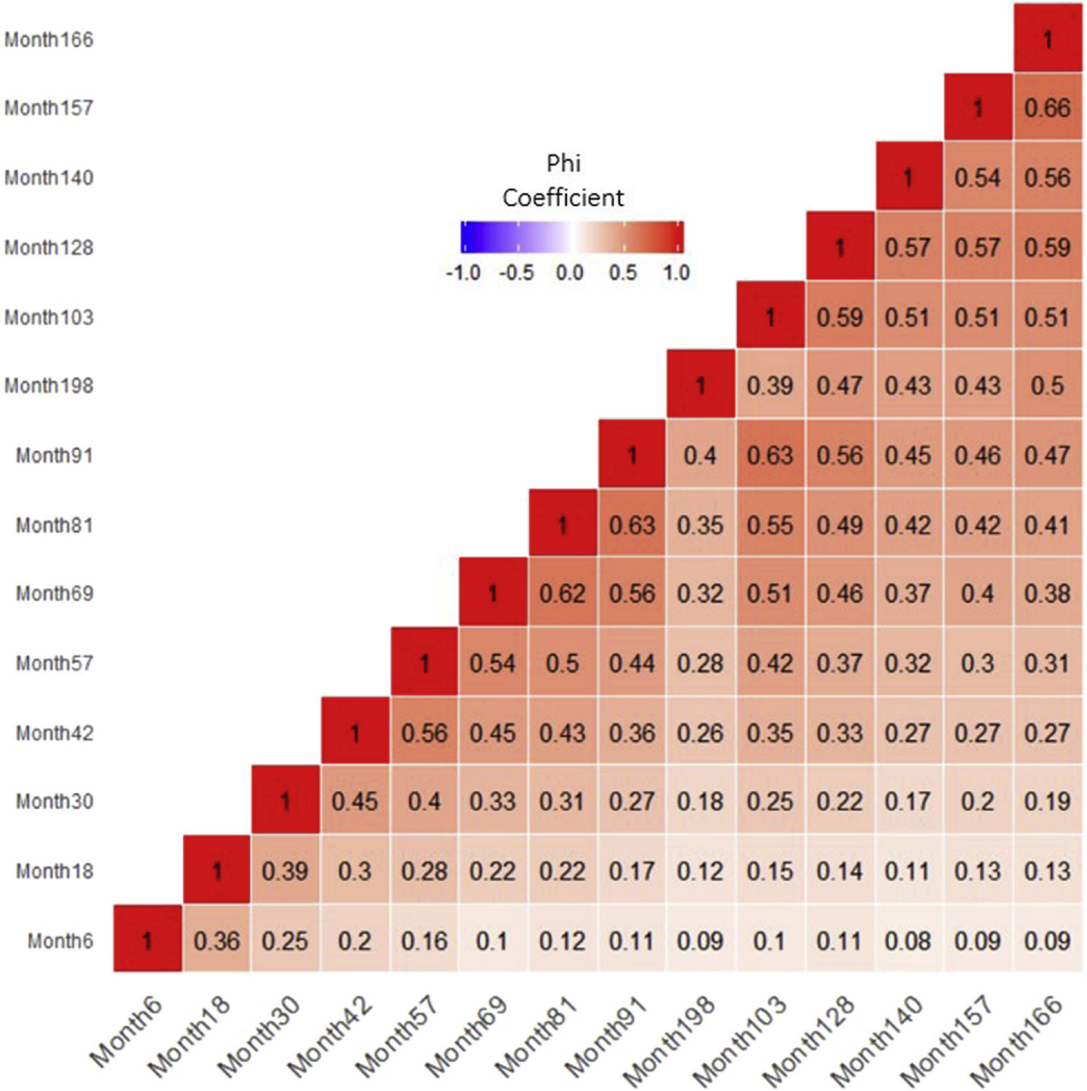
Heat map showing the phi coefficient of pairwise comparison between data collection points.

Fig E2 in this article's Online Repository at www.jacionline.org shows the correlations (phi coefficients) between wheeze reports at different time points. Time points close to each other were moderately correlated (eg, months 157 and 166 and months 81 and 91), suggesting that some of the adjacent time points convey similar information. To discard the noninformative data collection points, we performed stochastic evolutionary search through a genetic algorithm, which retained 8 informative time points (months 18, 42, 57, 81, 91, 140, 157, and 166), and 6 were dropped as uninformative (months 6, 30, 69,103, 128, and 198). Comparing the clustering of the models using 8 time points with the clustering from the model using the full data set showed a satisfactory level of agreement, with a Rand index and ARI of 82 and 64%, respectively (Table I).

**Table I:** Clustering summary of the LCA model fitted to the data subset (8 time points identified through a genetic algorithm search) and its comparison with the model fitted to the full data set (14 data collection points) based on 3167 participants with complete information on current wheeze recorded at 14 time points

Model characteristics		Variable selection (stochastic search)	
No. of classes	6	Selected time points (mo)	Univariate entropy
BIC	15,508	18	0.502
		42	0.581
Entropy	0.87	57	0.590
		81	0.578
Rand Index	0.82	91	0.588
ARI	0.64	140	0.549
Jaccard index	0.70	157	0.576
		166	0.582

### Latent transition probabilities with increasing numbers of classes

**Fig 2:**
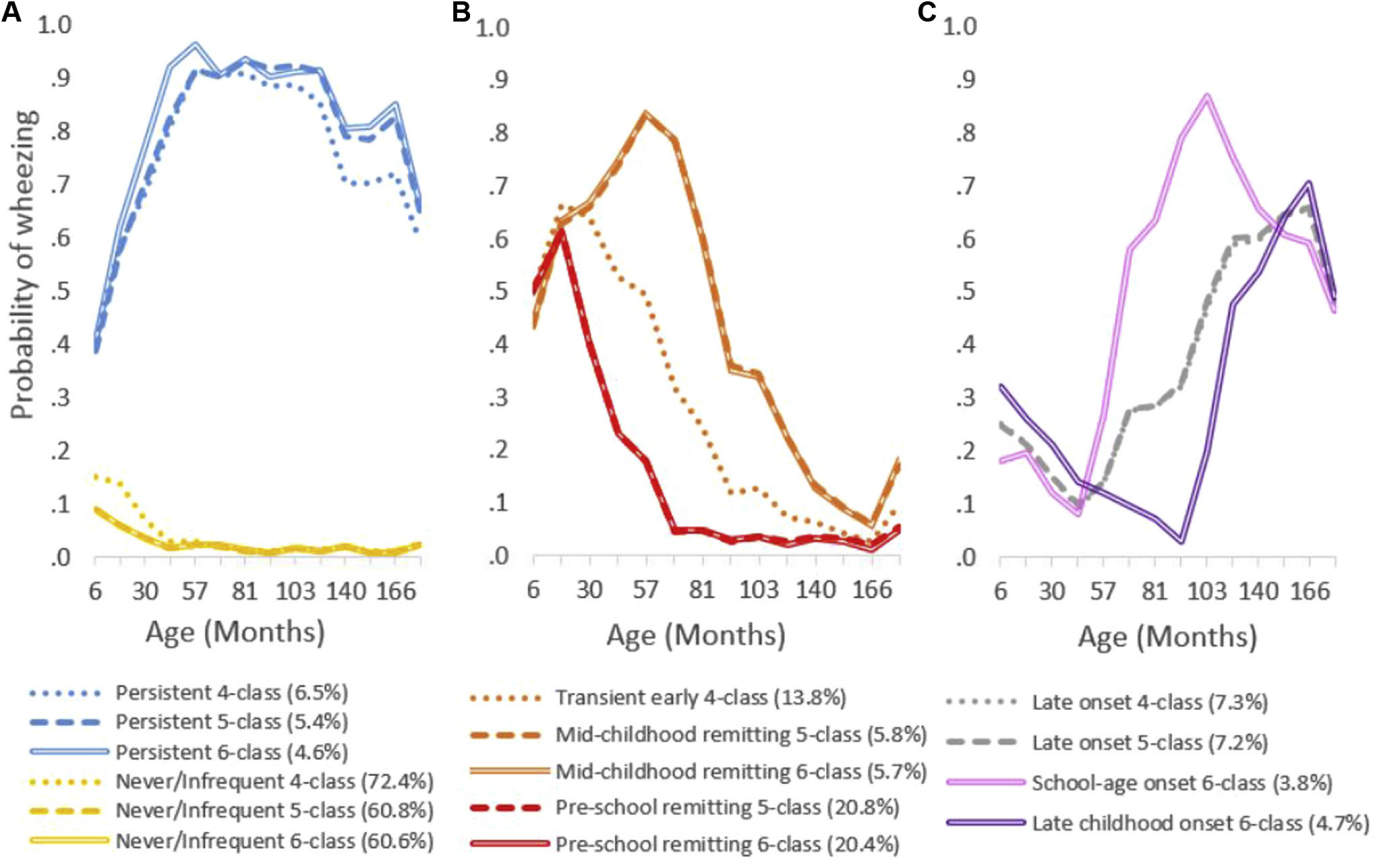
Estimated prevalence of wheezing for each wheeze phenotype in 4, 5, and 6 latent class solutions identified by using LCA.

**Fig 3:**
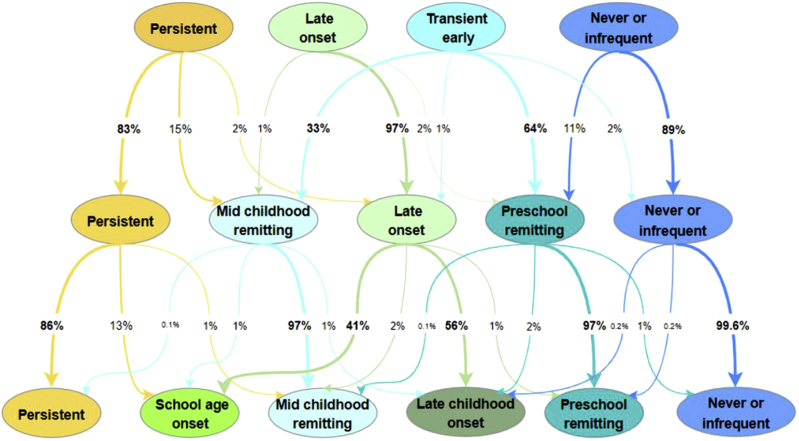
Assignment of children into distinct wheeze phenotypes over a sequence of latent class models with 4, 5, and 6 classes based on most likely class membership (cohort of 3167 children with complete reports of wheezing at 14 time points). *Ellipse nodes* show class membership (most likely phenotype), whereas *values along the arrow* represent the percentage of children moving from one class to another in models with an increasing number of classes.

**Fig E3:**
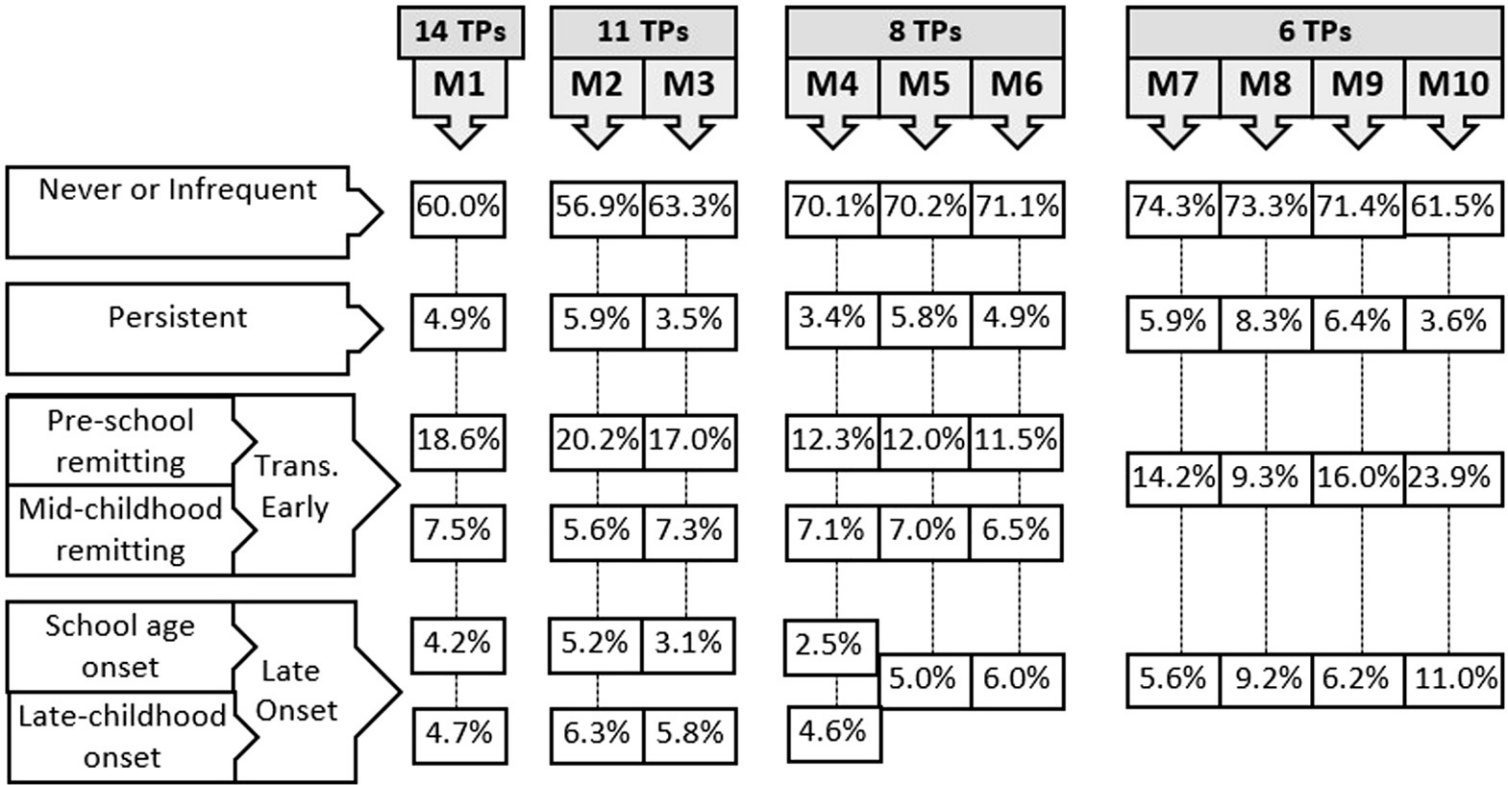
Optimal number, shape, and prevalence of wheeze phenotypes with different numbers of data collection points (6, 8, 11, and 14) based on children with at least 2 observations of wheeze.

**Fig E4:**
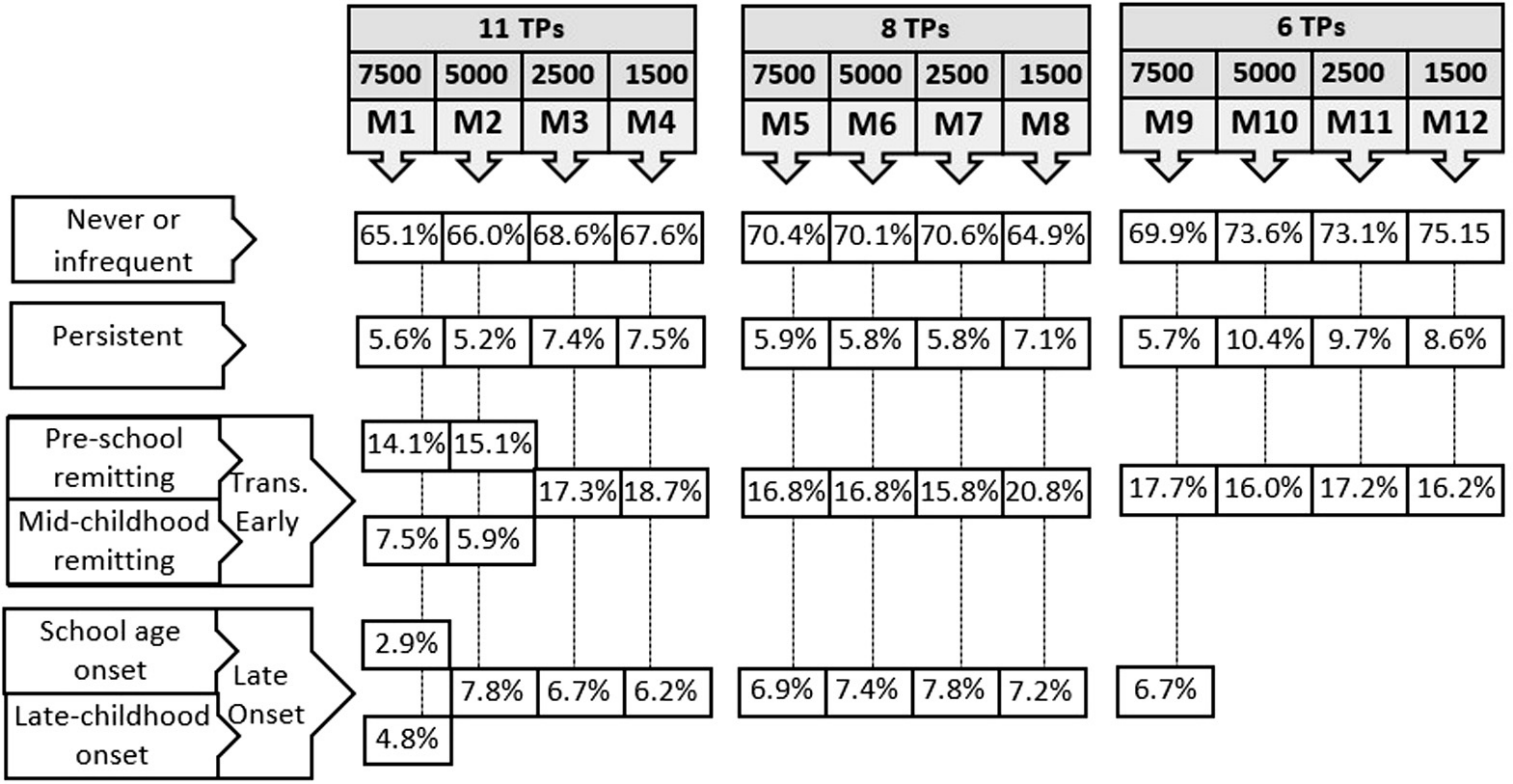
Optimal number, shape, and prevalence of wheeze phenotypes with combined effects of sample size and data collection frequency based on children with at least 2 observations of wheeze.

**Fig E5:**
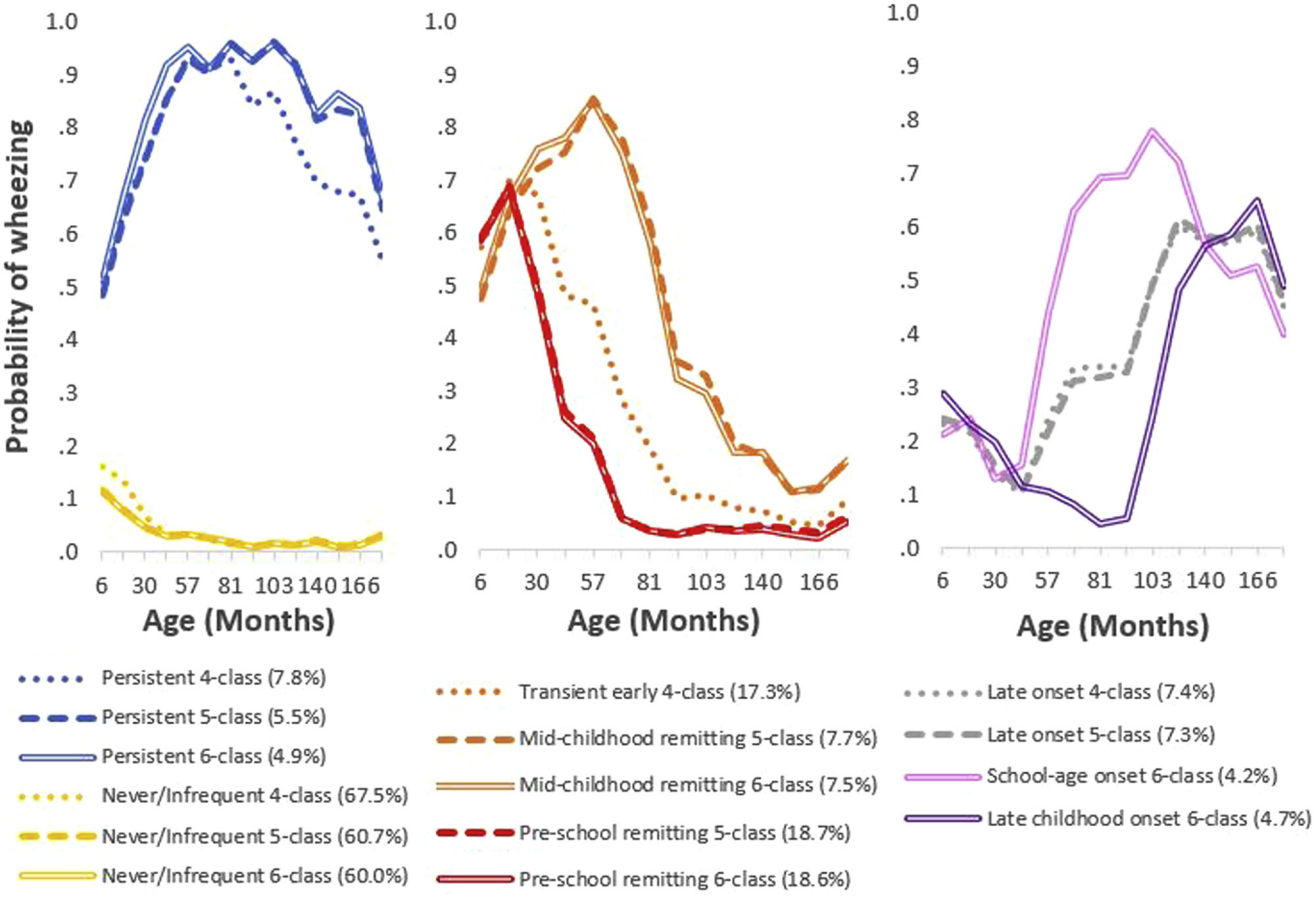
Estimated prevalence of wheeze for each wheeze phenotype in 4, 5, and 6 latent class solutions identified by using LCA based on 12,290 children with at least 2 observations of wheeze.

**Fig E6:**
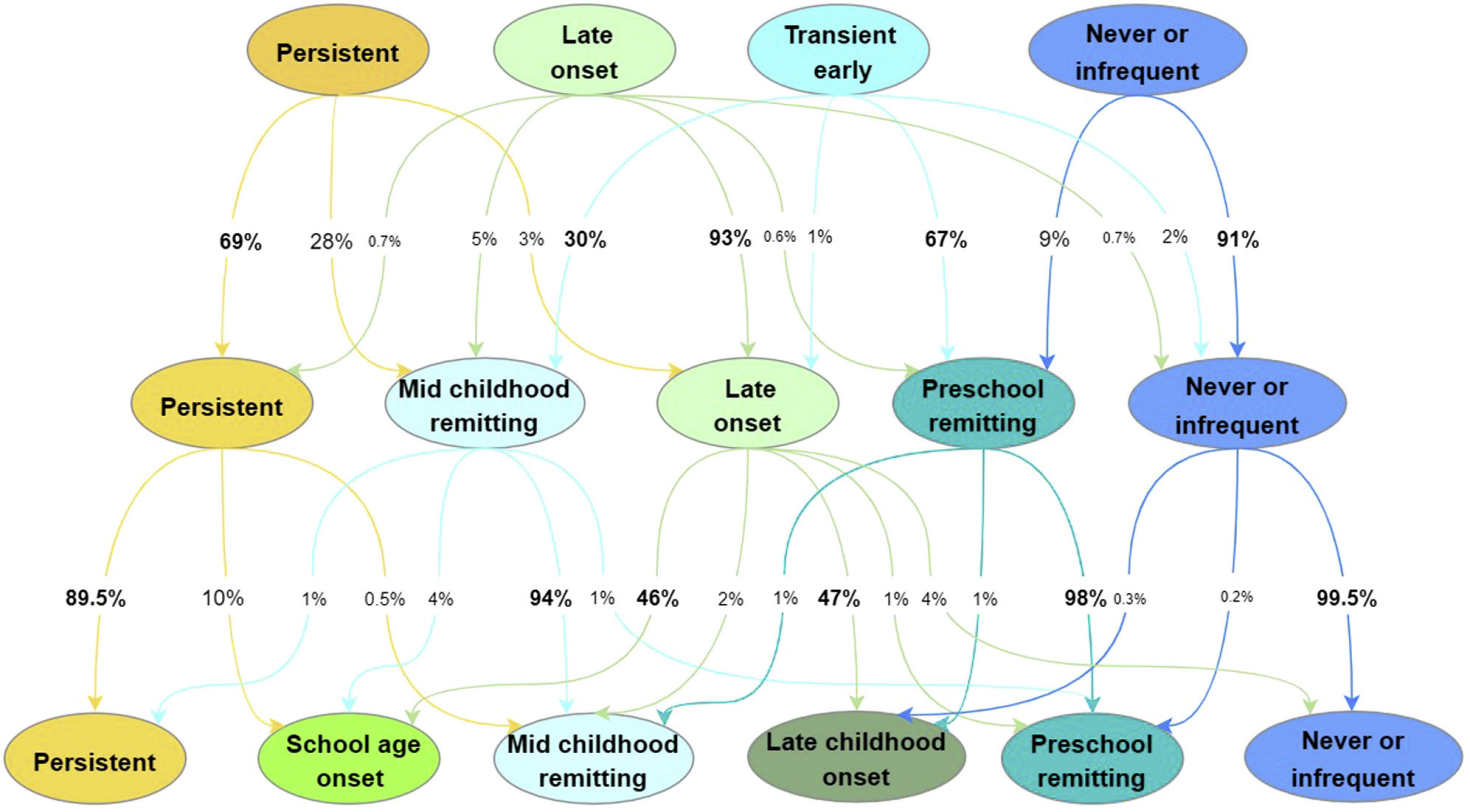
Assignment of children into distinct wheeze phenotypes over a sequence of latent class models with 4, 5, and 6 classes based on most likely class membership (12,290 children with at least 2 observations of wheezing). *Ellipse nodes* show class membership (most likely phenotype), whereas *values along the arrow* represent the percentage of children moving from one class to another in models with an increasing number of classes.

To understand how the trajectories and estimated phenotypes changed over a sequence of increasing numbers of classes and how children move from one class to another in models with increasing numbers of classes, we developed 3 LCA models with 4, 5, and 6 classes. Persistent and never/infrequent wheezing classes had similar patterns in all 3 models, with a slight decrease in estimated prevalence from a 4- to 6-class solution (Fig 2, *A*). With the addition of a fifth latent class, transient early wheezing was divided into 3 remitting classes (preschool and midchildhood resolution; Fig 2, *B*), whereas late-onset wheezing remained almost identical. The addition of a sixth class resulted in division of late-onset wheezing into 2 similarly sized subgroups (school-age and late-childhood onset; Fig 2, *C*). We then assigned participants to the most likely phenotype based on the maximum membership probability and calculated transition probabilities reflecting the proportion of participants moving from one phenotype to another when the number of phenotypes increased from 4 up to 6. Fig 3 shows whether members of distinct phenotypes remained in the same phenotype or shift into another one (either existing or newly formed) with increasing numbers of phenotypes. The figure also demonstrates from where the intermediate phenotypes arise and which phenotypes become separated or remain undivided with increasing numbers of latent classes. The results based on analysis of participants with incomplete reports of wheezing (12,290 participants with ≥2 responses to questionnaires about wheezing) did not materially differ from those obtained among children with a complete data set and are presented in Fig E1, Fig E3, Fig E4, Fig E5, Fig E6 in this article's Online Repository at www.jacionline.org.

### Asthma and lung function in adulthood in patients with different wheeze phenotypes

Of 3797 participants who attended follow-up at age 23 to 24 years, 1492 had complete reports of wheezing (14 points), of whom 240 (16%) reported current asthma; 1345 had valid lung function. The proportion of subjects with current asthma was greatest in the persistent wheeze phenotype (99.7%, Table II). In the 2 early-onset transient phenotypes, the proportion of asthmatic patients was significantly greater in midchildhood-remitting (60.4%) compared with the preschool-remitting (6.4%) phenotypes (mean difference, 0.5; 95% CI, 0.40-0.68; *P* < .0001). In the 2 late-onset phenotypes the proportion of asthmatic patient was significantly greater in the school-age onset (88.4%) compared with late-childhood onset (68.1%) phenotypes (mean difference, 0.20; 95% CI, 0.05-0.36; *P* < .02). Prebronchodilator and postbronchodilator lung function differed significantly across phenotypes (*P* = .005 and *P* = .04, respectively, ANOVA) and was significantly less in the persistent wheezing and early-onset preschool remitting wheeze phenotypes compared with the never/infrequent wheeze phenotype, with little evidence of differences between other phenotypes (Table III and see Table E1, Table E2, Table E3 in this article's Online Repository at www.jacionline.org). The preschool-onset remitting phenotype mostly overlapped with no asthma (94%), but prebronchodilator and postbronchodilator lung function at age 24 years was significantly less in this class compared with the never/infrequent wheeze phenotype.

**Table II:** Proportion of asthmatic patients at age 23 to 24 years in each phenotype

Wheeze phenotypes, 0-16½ y	Self-reported asthma ever		Current asthma at age 23 y		Asthma medication use at age 23 y	
	No. of asthmatic patients/total	Percentage∗	No. of asthmatic patients/total	Percentage∗	No. of medication users/total	Percentage∗
Never infrequent	105/1111	9.4	50/985	5.1	33/985	3.3
Transient early						
Preschool remitting	54/355	15.1	19/295	6.4	9/295	3.2
Midchildhood remitting	72/95	75.3	30/49	60.4	14/49	29.5
Late onset						
School-age onset	56/61	91.3	38/43	88.4	25/43	58.3
Late-childhood onset	58/82	70.0	38/55	68.1	25/55	45.3
Persistent wheeze	81/82	98.5	65/65	99.7	53/65	82.1

∗ The percentage is estimated from weighted cross-tabulations.

**Table III:** Lung function at age 24 years by wheeze phenotype (restricted to 1343 participants with FEV_1_ percent predicted data and 1351 with FEV_1_/FVC data)

Wheeze phenotypes, 0-16½ y	Baseline lung function at 24 y				Postbronchodilator lung function at 24 y			
	FEV_1_ (% predicted)		FEV_1_/FVC ratio		FEV_1_ (% predicted)		FEV_1_/FVC ratio	
	No.	Mean (SD)	No.	Mean (SD)	No.	Mean (SD)	No.	Mean (SD)
Never infrequent	1004	95.0 (11.7)	1009	0.84 (0.06)	830	97.9 (11.7)	834	0.86 (0.06)
Transient early								
Preschool remitting	329	93.4 (11.4)	330	0.82 (0.07)	274	96.8 (10.9)	275	0.85 (0.06)
Midchildhood remitting	89	93.5 (11.4)	91	0.82 (0.06)	71	97.5 (11.8)	73	0.84 (0.05)
Late onset								
School-age onset	61	95.4 (11.2)	61	0.81 (0.08)	47	100.8 (10.8)	47	0.86 (0.06)
Late-childhood onset	79	94.0 (12.1)	80	0.82 (0.07)	62	98.7 (10.8)	63	0.85 (0.05)
Persistent wheeze	80	91.6 (12.4)	80	0.79 (0.09)	59	96.5 (11.1)	59	0.83 (0.07)

*FVC*, Forced vital capacity.

## Discussion

### Key results

Our results suggest that the number and nature of wheeze phenotypes from infancy to adolescence identified by using LCA are dependent on several factors, including sample size, frequency, timing and distribution of data collection time points, model dimensionality, and the combination of these factors. Transition analysis revealed that subjects assigned to the never or persistent wheeze phenotypes tend to stay in these phenotypes, whereas most of the switching goes on in the intermediate classes. Given the strong interplay between the birth cohort design (including the number of participants, data collection frequency, and distribution) and the optimal number of phenotypes identified by means of developmental trajectory modeling, care should be taken when interpreting wheeze phenotypes emerging from small studies with few data collection points. When the sample size is small, a wheeze phenotype that exists in the population might be unidentifiable, whereas excessive data collection can result in identification of trivial or clinically irrelevant phenotypes. In general, increasing data collection frequency helps detect more complex structure and larger numbers of phenotypes by capturing less frequently observed subgroups. However, it also increases the risk of violating the fundamental assumption of LCA modeling where indicator variables (eg, presence/absence of wheezing at subsequent ages) are independent of each other. When frequent data collection and large sample sizes are not obtainable, collecting data at critical time points might help counterbalance the effects of suboptimal conditions (eg, smaller sample size and infrequent data collection). In our study the time points that proved most informative in distinguishing wheeze phenotypes were months 18, 42, 57, 81, 91, 140, 157, and 166.

### Limitations

There are several limitations to our findings. Despite latent models' usefulness in disentangling disease complexity, 1 unresolved issue in the application of LCA is that there is not one commonly accepted statistical indicator for deciding on the number of subgroups in a study population. The limitation of this study is that we do not know how many true phenotypes there are, and we assumed that the classification obtained on the largest sample and using all time points corresponded to the best available approximation of the “true classification.” In the absence of clear statistical requirements for identifying clinically important groups of small size, validation of the phenotypes with late asthma outcomes provides the only clues about their clinical relevance. However, we acknowledge that in our study information on asthma and lung function measures at age 23 to 24 years was available for approximately 45% of participants used to derive wheeze phenotypes.

Another limitation is that we could only vary conditions using the sampling framework that was available to us, which was fixed by the study design, and therefore this analysis has limited direct application to other studies that have used different sampling frames. We also acknowledge that the definition of current wheezing, which we used in our models, is based on parental reporting using validated questionnaires (as in most other epidemiologic studies) and that this might lead to overestimation of the true prevalence.^28^

As most previous studies, we used information on current wheeze for our modeling. It is possible that a more holistic examination of other features (eg, frequency and severity of wheezing) and/or other symptoms (eg, cough, atopic dermatitis, and rhinitis)^22^ and lung function^29^ might allow better distinction of the underlying pathophysiologic mechanisms.

The key advantage of our study is the large sample size with complete data on wheezing collected frequently and prospectively. Another advantage is that participants were followed from birth to late adolescence, covering a longer period compared with most prior studies.^1^, ^13^, ^18^, ^19^, ^30^

Finally, it is worth noting that subtypes discovered by using data-driven methods are not observed but are latent by nature and ideally should not be referred to as “phenotypes” (ie, observable characteristics). However, because the term “phenotype” has been used in this context for more than a decade, we have maintained this nomenclature.

### Interpretation

A number of previous studies (including our own) embarked on identifying wheeze phenotypes from birth to mid–school age (summarized in Table E4 in this article's Online Repository at www.jacionline.org). However, the inconsistency of findings has led to a debate on the validity and clinical value of phenotyping studies,^10^, ^31^, ^32^ hampering the discovery of pathophysiologic endotypes and translation into clinically actionable insights. The 4 phenotypes of persistent, never, transient early, and late-onset wheezing have been long postulated in descriptive^2^ and data-driven^33^ studies. We found that when the sample size is relatively small, a particular wheeze phenotype that exists in the population might be undetectable. Therefore relatively smaller sample size in some studies might have contributed to the inability to detect intermediate wheeze phenotypes with a relatively low prevalence. Using more time points allowed identification of less common phenotypes (<5% frequency) by increasing possible response patterns. When the data collection was frequent (>11 time points), a sample size of approximately 2500 was found to be sufficiently large to distinguish 6 phenotypes. However, even a larger sample size of 3167 might be insufficient to detect uncommon phenotypes (<5% frequency) under certain conditions (eg, data collection points <11). Our findings suggest that increasing data collection frequency might help compensate for a modest sample size in phenotype identification. In line with this finding, Depner et al^30^ identified an intermediate phenotype in the PASTURE cohort that existed during the first 6 years of life by using a similar sample size but more data collection points than those used in the TCRS.^2^ However, the selection of follow-up points needs careful thought. Our analyses have shown that although adding more time points to the latent model increased the number of identified phenotypes with distinguishable interpretations, in some cases the same number of randomly selected data collection points resulted in a different optimal solution. This suggests that the timing and distribution of follow-up is important and that there might be critical data collection points that are more informative than others. A variable selection method that we applied to the data identified 6 time points that were not carrying additional useful information (months 6, 30, 69, 103, 128, and 198).

The proportion of asthmatic patients was greatest in the persistent wheeze phenotype (98.5%), and subjects in this phenotype had diminished prebronchodilator and postbronchodilator lung function (at the time of maximally attained physiologic lung function plateau^29^) compared with all other phenotypes. The proportion of asthmatic patients differed between intermediate phenotypes (15.1% and 75.3% in 2 transient early phenotypes, preschool remitting and midchildhood remitting, respectively; 91.3% and 70.0% in 2 late-onset phenotypes, late childhood and school-age onset, respectively). These findings suggest that all phenotypes are distinct and that this might be a true classification. However, we acknowledge that the observed associations might also be a proxy of severity.

The preschool-onset remitting phenotype mostly overlapped with no asthma (94%), but the prebronchodilator and postbronchodilator lung function at age 24 years was significantly lower in this class compared with the never/infrequent wheeze phenotype. Although this can be seen as a contradiction, we would stress that diminished lung function does not equate to asthma.^29^ There is evidence that early transient wheezing is associated with low lung function^34^, ^35^, ^36^, ^37^; as lungs/airways grow, the symptoms regress, but lung function impairment can persist. In TCRS the lowest infant lung function test values were associated with low lung function at 22 years,^38^ and therefore early wheezing that remits might be a marker of low lung function in early life that persists to adulthood but without the development of airway inflammation or asthma.

In conclusion, our findings add to the understanding of childhood wheeze phenotypes by extending the knowledge on potential causes of variability in classification of wheezing. Sample size, frequency, and timing of data collection have a major influence on the number and type of phenotypes identified by using data-driven techniques. Our results, which include information on the most informative follow-up points, are important to interpret (or reanalyze) existing studies and to inform better design of future cohorts. However, we wish to note that these data collection points should not be regarded as absolute; rather, they should be treated as relative values with respect to our population and considerations for investigators when designing future studies.

**Key messages**

• The number and nature of wheeze phenotypes identified by using LCA are dependent on the sample size, frequency, timing and distribution of data collection time points; model dimensionality; and combinations of these factors.
• Not all data collection points carry useful information in distinguishing wheeze phenotypes.

## Figures and Tables

**Table E1:** Differences in lung function (FEV_1_ percent predicted) at 24 years between wheeze phenotypes: ANOVA and Tukey HSD test of pairwise comparisons

ANOVA										
Variation source	*df*		Sum square		Mean square		F value		Pr(>F)	
	Pre-BD FEV_1_ % predicted	Post-BD FEV_1_ % predicted	Pre-BD FEV_1_ % predicted	Post-BD FEV_1_ % predicted	Pre-BD FEV_1_ % predicted	Post-BD FEV_1_ % predicted	Pre-BD FEV_1_ % predicted	Post-BD FEV_1_ % predicted	Pre-BD FEV_1_ % predicted	Post-BD FEV_1_ % predicted
Phenotypes	5	5	2,243	1,463	448.6	292.6	3.313	2.252	0.005561	0.04709
Residual	1,651	1,351	223,565	175,521	135.4	129.9				

Tukey HSD test						
Pairwise comparison of wheeze phenotypes	Mean Differences		Significant (*P* adjusted< .05)?		95% CI of differences (lower, to upper)	
	Pre-BD FEV_1_ % predicted	Post-BD FEV_1_ % predicted	Pre-BD FEV_1_ % predicted	Post-BD FEV_1_ % predicted	Pre-BD FEV_1_ % predicted	Post-BD FEV_1_ % predicted
Midchildhood remitting AND late-childhood onset	−0.66	−1.61	No	No	−5.82 to 4.49	−7.27 to 4.05
Never/infrequent AND late-childhood onset	0.69	−1.34	No	No	−3.11 to 4.50	−5.52 to 2.84
Preschool remitting AND late-childhood onset	−1.59	−3.11	No	No	−5.78 to 2.60	−7.69 to 1.47
Persistent AND late-childhood onset	−3.26	−3.41	No	No	−8.44 to 1.93	−9.26 to 2.44
School-age onset AND late-childhood onset	1.51	1.70	No	No	−4.11 to 7.12	−4.45 to 7.86
Never/infrequent AND midchildhood remitting	1.36	0.27	No	No	−2.40 to 5.12	−3.85 to 4.39
Preschool remitting AND midchildhood remitting	−0.93	−1.50	No	No	−5.08 to 3.22	−6.03 to 3.03
Persistent AND midchildhood remitting	−2.59	−1.80	No	No	−7.75 to 2.56	−7.61 to 4.00
School-age onset AND midchildhood remitting	2.17	3.31	No	No	−3.42 to 7.76	−2.80 to 9.43
**Preschool remitting AND never/infrequent**	−2.29	−1.77	**Yes**	No	−4.55 to −0.02	−4.20 to 0.66
**Persistent AND never/infrequent**	−3.95	−2.07	**Yes**	No	−7.76 to −0.15	−6.45 to 2.30
School-age onset AND never/infrequent	0.81	3.04	No	No	−3.56 to 5.18	−1.73 to 7.82
Persistent AND preschool remitting	−1.66	−0.30	No	No	−5.86 to 2.52	−5.06 to 4.46
School-age onset AND preschool remitting	3.10	4.81	No	No	−1.61 to 7.81	−0.32 to 9.94
School-age onset AND persistent	4.77	5.12	No	No	−0.85 to 10.38	−1.17 to 11.4

Significant differences are in boldface.

*HSD*, Honestly significant difference.

**Table E2:** Differences in lung function (FEV_1_/FVC ratio) at 24 years between wheeze phenotypes: ANOVA and Tukey HSD test of pairwise comparisons

ANOVA										
Variation source	*df*		Sum square		Mean square		F value		Pr(>F)	
	Pre-BD FEV_1_/FVC ratio	Post-BD FEV_1_/FVC ratio	Pre-BD FEV_1_/FVC ratio	Post-BD FEV_1_/FVC ratio	Pre-BD FEV_1_/FVC ratio	Post-BD FEV_1_/FVC ratio	Pre-BD FEV_1_/FVC ratio	Post-BD FEV_1_/FVC ratio	Pre-BD FEV_1_/FVC ratio	Post-BD FEV_1_/FVC ratio
Phenotypes	5	5	0.3269	0.1527	0.06539	0.03054	14.51	8.33	6.2E-14	9.0E-08
Residual	1600	1358	7.482	4.977	0.00451	0.00366				

Tukey HSD test						
Pairwise comparison of wheeze phenotypes	Mean Differences		Significant (*P* adjusted < .05)?		95% CI of differences (lower to upper)	
	Pre-BD FEV_1_/FVC ratio	Post-BD FEV_1_/FVC ratio	Pre-BD FEV_1_/FVC ratio	Post-BD FEV_1_/FVC ratio	Pre-BD FEV_1_/FVC ratio	Post-BD FEV_1_/FVC ratio
Midchildhood remitting AND late-childhood onset	0.01	−0.01	No	No	−0.02 to 0.04	−0.04 to 0.02
Never/infrequent AND late-childhood onset	0.02	0.01	No	No	0.00 to 0.04	−0.02 to 0.03
Preschool remitting AND late-childhood onset	0.00	−0.01	No	No	−0.03 to 0.02	−0.04 to 0.01
Persistent AND late-childhood onset	−0.03	−0.03	No	No	−0.06 to 0.00	−0.06 to 0.00
School-age onset AND late-childhood onset	0.00	0.00	No	No	−0.04 to 0.03	−0.03 to 0.03
Never/infrequent AND midchildhood remitting	0.01	0.02	No	No	−0.01 to 0.04	0.00 to 0.04
Preschool remitting AND midchildhood remitting	−0.01	0.00	No	No	−0.04 to 0.01	−0.02 to 0.02
Persistent AND midchildhood remitting	−0.03	−0.02	**Yes**	No	−0.06 to 0.00	−0.05 to 0.01
School-age onset AND midchildhood remitting	−0.01	0.01	No	No	−0.04 to 0.02	−0.02 to 0.04
**Preschool remitting AND never/infrequent**	−0.03	−0.02	**Yes**	**Yes**	−0.04 to −0.01	−0.03 to −0.01
**Persistent AND never/infrequent**	−0.05	−0.04	**Yes**	**Yes**	−0.07 to −0.03	−0.06 to −0.01
School-age onset AND never/infrequent	−0.03	−0.01	**Yes**	No	−0.05 to 0.00	−0.03 to 0.02
Persistent AND preschool remitting	−0.02	−0.02	No	No	−0.05 to 0.00	−0.04 to 0.01
School-age onset AND preschool remitting	0.00	0.01	No	No	−0.03 to 0.03	−0.01 to 0.04
School-age onset AND persistent	0.02	0.03	No	No	−0.01 to 0.05	0.00 to 0.06

Significant differences are in boldface.

*FVC*, Forced vital capacity; *HSD*, honestly significant difference.

**Table E3:** FEV_1_ reversibility at age 24 years by phenotype (restricted to 1364 participants with FEV_1_ reversibility data)

Wheeze phenotypes, 0-16½ y	FEV_1_ reversibility at 24 y		*P* value∗	Percent positive FEV_1_ reversibility (optional)
	No.	Mean (SD)		
Never-infrequent	898	2.87 (6.2)	Baseline	80.0
Transient early				
Preschool remitting	224	4.02 (5.4)	0.104	81.3
Midchildhood remitting	69	4.03 (5.3)	0.627	84.1
Late onset				
School-age onset	49	4.94 (4.9)	0.171	89.8
Late-childhood onset	65	4.22 (5.4)	0.490	83.1
Persistent wheeze	59	6.29 (5.9)	0.0003	91.5

∗ Tukey test.

**Table E4:** Wheeze phenotypes from birth up to 9 years of age identified based on temporal pattern

Cohort	Sample Size	No. of time points	Years covered	No. of phenotypes
CCCEH^E7^	689	15	9	4
TUSCON^E8^	826	2	6	4
PASTURE^E9^	953	6	6	5
MAAS^E10^	1184	8	8	5
PIAMA^E11^	2810	8	8	5
ALSPAC^E11^	5760	8	8	6
ALSPAC^E12^	6265	7	7	6

*CCCEH*, Columbia Center for Children’s Environmental Health; *MAAS*, Manchester Asthma and Allergy Study; *PASTURE*, Protection against Allergy—Study in Rural Environments; *PIAMA*, Prevention and Incidence of Asthma and Mite Allergy; *TUCSON*, Tucson Children’s Respiratory Study.

## Methods

### Study design

This was an unselected birth cohort study.

### Setting

ALSPAC is based on the former administrative County of Avon, United Kingdom covering a population of approximately 0.9 million.

### Screening and recruitment

ALSPAC initially recruited 14,541 pregnant women residing in Avon, United Kingdom, with expected dates of delivery between April 1, 1991, and December 31, 1992. This initial number of pregnancies, known as the core sample, included mothers enrolled in ALSPAC and had either returned at least 1 questionnaire or attended a Children in Focus research clinic by July 19, 1999. These initial pregnancies had a total of 14,676 fetuses, resulting in 14,062 live births and 13,988 children who were alive at age 1 year. When the oldest children were approximately 7 years of age, an attempt was made to bolster the initial sample with eligible cases who had not joined the study originally. As a result, there are extra data available when considering variables collected from the age of 7 years onward. The number of new pregnancies not in the core sample, known as phase II and III enrollments, is 706 (452 and 254 recruited during phases II and III, respectively), resulting in an additional 713 children being enrolled. The phases of enrollment are described in more detail in the cohort profile paper.E1, E2 Therefore the total sample size for analyses using any data collected after the age of 7 years is 15,247 pregnancies, resulting in 15,458 fetuses with 14,775 live births and 14,701 alive children at 1 year of age.

### Spirometry

Spirometry was performed according to American Thoracic Society/European Respiratory Society guidelinesE3, E4 with a Vitalograph pneumotachograph system using animated incentive software (Spirotrac; Vitalograph, Maids Moreton, United Kingdom) in a dedicated research clinic by trained technicians. Calibration checks were performed with a standard 3-L calibration syringe, according to the manufacturer's instructions, at the start of each half-day clinic session. Subjects were seated with a nose clip in place and were asked to inhale to total lung capacity and then instructed to perform a forced expiration through a mouthpiece to residual volume. The test was repeated at intervals of 30 seconds until 3 technically acceptable traces were obtained from a maximum of 8 attempts. FEV_1_ and forced vital capacity were recorded, and data were expressed as FEV_1_ percent predicted and FEV_1_/forced vital capacity ratio.

### Definition of outcomes

*Current wheeze* was defined as a positive answer to the following question: “In the last 12 months has he/she had any periods when there was wheezing or wheezing with whistling on his/her chest when he/she breathed?”

*Current asthma* was defined as self-reported current asthma at 23 years based on asthma ever at age 22 years or greater together with current wheezing and/or current treatment: “Have you had any wheezing in the past 12 months?” and/or “Have you taken asthma medication in the last 12 months?”

*Asthma ever* was defined as a positive answer to the question “Have you ever had asthma?” at age 22 years or greater.

Study data were collected and managed with REDCap electronic data capture tools hosted at ALSPAC facilities.^E5^ Please note that the study Web site contains details of all the data that are available through a fully searchable data dictionary and variable search tool (http://www.bris.ac.uk/alspac/researchers/data-access/data-dictionary/).

### Statistical analysis

#### Measures of fit in LCA

An optimal model is defined as the free model that best fits the data. To assess model fit, we used (1) the BIC, a function of the likelihood that rewards parsimony, and (2) entropy, an assessment of model classification based on the posterior class membership probabilities. The BIC is an index used in Bayesian statistics to choose among a set of competing models; the model with the lowest BIC is preferred. Entropy is a measure of classification certainty that that ranges from 0 to 1, with values near 1 indicating a clear delineation of classes and values near 0 indicating low certainty in classification.

#### Selection of informative data collection points

A genetic algorithm was used to search for the optimal set of clustering variables (eg, time points) to distinguish wheezing subgroups by using the LCAvarsel R package.^E6^ During the search, multiple sets of clustering variables were considered at the same time; then, for each set, an LCA model was estimated on the clustering variables, and a regression/independence model was estimated on the nonclustering variables. Different sets were generated by various genetic operators, and the fittest subjects were selected. The fitness function was defined as the BIC of the joint distribution of both clustering and nonclustering variables, where clustering variables were modeled through an LCA model and nonclustering variables were modeled through multinomial logistic regression. Variable specific entropy contribution of each time point was used to assess how well individual time points identified latent classes. These univariate entropies varying between 0 and 1 were directly comparable with each other, with large values indicating the clear separation of classes.

The Rand index and ARI were used as similarity measures when comparing different clustering results. More specifically, the ARI was used to measure the level of agreement between 2 partitions, the model fitted to the data subset and the full data set. A larger Rand index and ARI means greater agreement between 2 partitions.
